# Evaluation of the long-term skeletal effect induced by teratogen 5-aza-2′deoxycytidine on offspring of high (C3H/HeJ) and low (C57BL/6J) bone mass phenotype mice

**DOI:** 10.1016/j.bonr.2018.05.005

**Published:** 2018-05-29

**Authors:** Deepak Kumar Khajuria, Maria Raygorodskaya, Eugene Kobyliansky, Yankel Gabet, Sahar Hiram Bab, Chen Shochat, Arkady Torchinsky, David Karasik

**Affiliations:** a The Musculoskeletal Genetics Laboratory, Azrieli Faculty of Medicine, Bar Ilan University, Safed, Israel; b Department of Anatomy & Anthropology, Sackler Faculty of Medicine, Tel Aviv University, Tel Aviv, Israel

**Keywords:** Bone loss, Genetic heterogeneity, Adult mice, Developmental origin of diseases

## Abstract

The long term skeletal effects of antenatal exposure to teratogen 5-deoxy-2′-cytidine (5-AZA) were studied using two inbred strains, C3H/HeJ (C3H, with inherently stronger bones) and C57Bl/6J (C57, with weaker bones). We previously reported that *in-utero* exposure to 5-AZA resulted in loss of bone quality in 3- and 6-mo-old C3H offspring. In this study, we further examined whether the long-term effects of an acute teratogenic exposure are still evident in older mice. Bone phenotypes of 12 mo-old mice exposed to a single injection of 5-AZA on day 10 of their mother's pregnancy were evaluated by micro-computed tomography and compared to the untreated controls.

The main observation of this study is that 5-AZA-induced loss of bone length was registered in 12-mo-old C57 and C3H males. As expected, we did not find differences in the 3rd lumbar vertebra since *in-utero* exposure to 5-AZA was shown to affect the limb buds but not the axial skeleton. Trajectory of changes in bone phenotypes from ages 3 mo through 6 mo to 12 mo was also compared; 5-AZA-exposed C57 males had consistently lower femoral length and trabecular BMD than age-matched controls. In summary, by characterizing teratogen-exposed C57 and C3H mice, we further confirmed that the adaptive response to antenatal insults continue into mid-life of the mice as well as there is a sex-specificity of these responses.

## 1 Introduction

Antenatal response to intrauterine stress (Oberbauer, 2015) is an inherent trait of an organism. In response to environmental stresses, early life compensatory mechanisms are critical for establishing skeletal strength (Jepsen, 2009). We previously reported that strain genetics influences the response to 5-deoxy-2′-cytidine (5-AZA) in C3H/HeJ (C3H) and C57Bl/6J (C57) mice and contemplated about the potential mechanisms of strain and sex effects observed in young/adult animals (Raygorodskaya et al., 2016). Our choice of 5-AZA was dictated by the fact that teratological studies in mice have revealed that 5-AZA injected on day 10 of gestation induces phocomelia (absence of the long bones of the limb) in a dose-dependent fashion (Torchinsky et al., 2012). This exposure time of 5-AZA approximately matched the very beginning of osteoblastogenesis (Maes et al., 2007). It was originally shown that 5-mo-old offspring of ICR mice exposed to a single injection of 5-AZA at a sub-threshold teratogenic dose on day 10 of pregnancy exhibit femoral trabecular microarchitecture (bone 3D structure assessed at high resolution) indicative of bone loss, while such a dose does not affect maternal homeostasis (Torchinsky et al., 2012).

The choice of C57 and C3H mouse strains was dictated by the fact that these two strains have been suggested for almost two decades as a model system to study genetics of osteoporosis (Chen & Kalu, 1999), where C3H represents a model of high bone mass phenotype, and C57 represents a model of low bone mass phenotype. In the present study we further examined whether single *in utero* exposure to 5-AZA has long-term effects, which continue into adulthood and mid-life. The mice exposed to a single injection of 5-AZA on day 10 *in utero* were evaluated by micro-computed tomography (μCT) at age 12 mo in comparison to non-exposed control mice. Elucidating whether antenatal and early-life stress affect the adult skeleton is highly relevant to our understanding of genome-by-environment impact on bone mineral density (BMD) throughout life.

## 2 Material and methods

### 2.1 Animals

Eight-week-old C57BL/6JRccHsd (C57) and C3H/HeJ (C3H) mice were obtained from Envigo (formerly Harlan Laboratories), Jerusalem, Israel (Raygorodskaya et al., 2016). Standard procedures were used for animal maintenance, feeding and breeding. 5-AZA (Sigma, St. Louis, MO, USA) was injected intraperitoneally on day 10 of gestation at 0.15 mg/kg (in 0.2 ml saline/20 g body weight). Control pregnant dams were injected with saline (0.2 ml/20 g body weight). Offspring were obtained from 6 C57 and 6 C3H dams exposed to 0.15 mg/kg 5-AZA and 4 C57 and 4 C3H control dams. At 3 and 6 mo of age, male and female offspring of treated and control mice were selected randomly for morphometric analysis using *ex vivo* μCT. Analyses of 3- and 6-mo old mice were reported (Raygorodskaya et al., 2016). The rest of the offspring were maintained until 12 months of age and then sacrificed. Left femora and 3rd lumbar vertebrae were collected from the offspring at the age of 12 mo, fixed in 3.7% formaldehyde in phosphate buffered saline (PBS) for 24 h and subsequently stored in 70% ethanol until scanning (5 males and 5 females of control and 5-AZA treated C57 and C3H mice tested). At sacrifice, mice were weighted using electronic balance scales BB-3100 (Table 1). Animal experiments and maintenance were conducted at the Bar-Ilan University/Faculty of Medicine animal facility and approved by the IACUC of Bar Ilan University, Israel.

**Table 1:** Average body weight (g) of different groups of mice at 12 mo of age (presented as group mean ± S.E.).

Strain	C3H/HeJ				C57BL/6 J			
Sex	Males		Females		Males		Females	
Treatment	Treated	Untreated	Treated	Untreated	Treated	Untreated	Treated	Untreated
Weight	33.3 ± 0.8	35.8 ± 0.7	34.2 ± 0.5	35.9 ± 0.9	35.4 ± 0.4	37.3 ± 0.9	36.3 ± 0.6	37.7 ± 0.5
*p*-value	0.0205		0.403		0.0122		0.523	

Student's *t*-test with Bonferroni correction; statistical significance was set at *p* ≤ 0.0062.

### 2.2 Micro-computed tomography (μCT)

μCT analysis was performed using μCT50 system (Scanco Medical AG, Brüttisellen, Switzerland) as described in (Raygorodskaya et al., 2016). In the entire intact femur we measured volumetric bone mineral density (vBMD) and axial length using the calibrated μCT software (Scanco, uct_evaluation v6.5-3). In the femoral cortical bone, we used a 1-mm-height diaphyseal segment extending distally from the mid-shaft. The femoral trabecular traits were examined in the distal half (1.5 mm height) of the distal metaphysis (dVOI). The following cortical and trabecular parameters were measured in femora (Bouxsein et al., 2010; Manolagas & Kronenberg, 2014): diaphyseal (Dia.Dia) and medullary cavity (Med.Dia) diameters, cortical thickness (Ct.Th), tissue mineral density (TMD), trabecular bone volume fraction (bone volume/tissue volume ratio, BV/TV), trabecular number (Tb.N), trabecular thickness (Tb.Th) and trabecular separation (Tb.Sp). In the 3rd lumbar vertebrae we measured the following trabecular parameters: BV/TV, Tb.N, Tb.Th and Tb.Sp.

### 2.3 Statistical analysis

Parameters reported by μCT as well as the weight of the offspring were analyzed by Student's *t*-test with Bonferroni correction reflecting multiple-testing adjustment (2 strains × 2 sexes × 2 exposures [control and treated]). Statistical significance was set at *p* ≤ 0.0062.

## 3 Results

### 3.1 Body weight

Weight of 12-mo-old offspring is presented in Table 1. Weight in treated 12-mo-old C3H and C57 males appeared lower than that of age-matched controls (non-significant).

### 3.2 Bone microarchitecture: 12 mo-old C57 offspring

Femoral length in treated 12-mo-old C57 males was significantly lower from that of age-matched controls (*p* = 0.0041, Table 2). The analysis of trabecular bone parameters suggested that the treated C57 males demonstrated significantly lower BV/TV and Tb.N in comparison to the age-matched controls (*p* < 0.0001, Table 2). In the cortical bone, no significant differences were observed (all *p* > 0.0062). No significant changes in vertebral bone microarchitecture were observed in treated C57 males and females as compared to age-matched controls (data not shown).

**Table 2:** Bone morphometry and density of the femur measured by μCT at age 12 mo (presented as group mean ± S.E.).

			Full bone		Cortical				Trabecular			
			Length [mm]	vBMD [mg HA/cm^3^]	Ct.Th [mm]	Dia.Dia [mm]	Med.Dia [mm]	TMD [mg HA/cm^3^]	BV/TV [%]	Tb.Th [mm]	Tb.N [mm^−1^]	Tb.Sp. [mm]
C57	Males	Untreated	15.60 ± 0.03	364.70 ± 28.32	0.13 ± 0.01	1.49 ± 0.01	1.22 ± 0.01	847.03 ± 12.45	4.15 ± 0.40	0.047 ± 0.001	0.87 ± 0.07	0.392 ± 0.010
		Treated	15.26 ± 0.08	344.40 ± 16.56	0.14 ± 0.01	1.51 ± 0.01	1.23 ± 0.01	864.18 ± 11.96	1.78 ± 0.20	0.045 ± 0.003	0.40 ± 0.05	0.431 ± 0.049
		*p*-Value	0.0041⁎	0.5533	0.4996	0.1950	0.4996	0.3496	0.0007⁎	0.5447	0.0001⁎	0.4579
	Females	Untreated	15.49 ± 0.05	378.06 ± 10.14	0.13 ± 0.01	1.52 ± 0.04	1.27 ± 0.04	926.81 ± 25.18	1.25 ± 0.41	0.053 ± 0.009	0.23 ± 0.06	0.564 ± 0.054
		Treated	15.32 ± 0.18	357.45 ± 21.67	0.13 ± 0.01	1.63 ± 0.02	1.36 ± 0.03	867.01 ± 32.57	0.90 ± 0.20	0.046 ± 0.002	0.20 ± 0.06	0.652 ± 0.071
		*p*-Value	0.3894	0.4141	1.000	0.0393	0.1096	0.1844	0.4650	0.4695	0.7328	0.3528
C3H	Males	Untreated	16.01 ± 0.05	704.77 ± 33.77	0.30 ± 0.01	1.64 ± 0.02	0.91 ± 0.06	1078.2 ± 13.61	9.28 ± 1.81	0.056 ± 0.001	1.67 ± 0.34	0.249 ± 0.027
		Treated	15.54 ± 0.06	669.54 ± 22.61	0.30 ± 0.02	1.60 ± 0.03	0.97 ± 0.02	1049.7 ± 21.69	6.60 ± 1.29	0.054 ± 0.005	1.22 ± 0.21	0.268 ± 0.006
		*p*-Value	0.0003⁎	0.4113	1.000	0.2995	0.3706	0.2980	0.2032	0.7051	0.2988	0.5115
	Females	Untreated	16.34 ± 0.08	892.10 ± 11.28	0.38 ± 0.01	1.50 ± 0.01	0.68 ± 0.03	1164.8 ± 81.23	10.41 ± 6.38	0.075 ± 0.003	1.41 ± 0.91	0.40 ± 0.01
		treated	15.96 ± 0.07	852.19 ± 19.36	0.45 ± 0.02	1.52 ± 0.01	0.62 ± 0.03	1177.5 ± 15.67	9.96 ± 0.78	0.079 ± 0.001	1.27 ± 0.09	0.42 ± 0.01
		*p*-Value	0.0072	0.1127	0.0140	0.1950	0.1950	0.8818	0.2415	0.2415	0.8821	0.1950

⁎ Statistically-significant differences (Student's *t*-test with Bonferroni correction; statistical significance was set at *p* ≤ 0.0062).

### 3.3 Bone microarchitecture: 12 mo-old C3H offspring

Femoral length in treated 12-mo-old C3H mice was significantly lower than that of age-matched controls (*p* < 0.0003 and 0.0072 for males and females, Table 2). Treated C3H males demonstrated lower BV/TV and Tb.N in comparison to the age-matched controls but the difference was not statistically significant (Table 2). In the cortical bone, no significant differences were observed (all *p* > 0.0062). No significant changes in vertebral bone microarchitecture were observed in treated C3H males and females as compared to age-matched controls (data not shown).

**Fig. 1:**
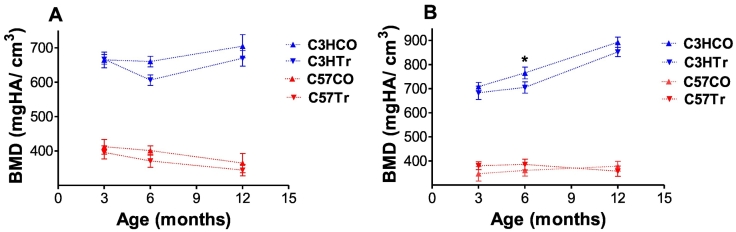
Trajectories of change in bone mineral density (BMD). A, males; B, females; CO – control; Tr, treated (comparison of TR and CO within strain and age: * *t*-test *p*-value <0.05).

Data for 3- and 6-mo mice are from Raygorodskaya et al. (2016).

**Fig. 2:**
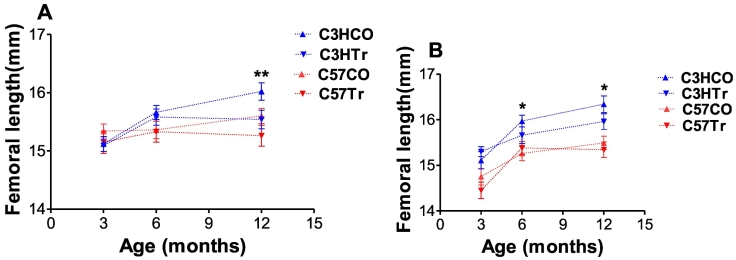
Trajectories of change in femoral length. A, males; B, females; CO – control; Tr, treated (comparison of TR and CO within strain and age: * t-test *p*-value <0.05; ** t-test *p*-value <0.01).

Data for 3- and 6-mo mice are from Raygorodskaya et al., (2016).

### 3.4 Change in bone characteristics between 3, 6 and 12 mo-old

In order to find out whether genome-by-environment interaction affected the bone traits between the three studied age points, we plotted strain or sex explained difference between 3-mo, 6-mo and 12-mo-old values in measured bone parameters. We revealed increase in femoral length as well as BMD from 3 to 12 mo-old in C3H (male & females) untreated controls as compared to 5-AZA treated male and female C3H mice. The untreated groups did behave in accordance to the published literature (*e.g.* at age 12 mo, BMD in C57 males was 378.1 ± 10.1 and in untreated C3H males, 704.8 ± 33.8, Table 2). While also increasing with age, femoral length and BMD in treated C57 male and C3H male and female offspring was lower from that of age-matched controls (Fig. 1, Fig. 2). In treated C3H females, we revealed decreased femoral BMD only at age 6 mo. Femoral lengths were significantly lower in treated C3H males and females at age 12 months. No significant changes in BMD or femoral length were observed in treated C57 males and females as compared to age-matched controls (Fig. 1, Fig. 2). Additional bone parameters are shown in Supplementary Fig. 1.

## 4 Discussion

We previously reported that the skeletal response to intrauterine stress in the 3- and 6-mo-old offspring's femora differed by strain (Raygorodskaya et al., 2016). *In-utero* exposure to 5-AZA, resulted in loss of bone quality in 6-mo-old C3H offspring. The main observation of the original study was that the adaptive response to antenatal insults (5-AZA) may be stronger in offspring inherently exhibiting a low bone mass phenotype (C57) than in offspring inherently exhibiting a high bone mass phenotype (C3H)(Raygorodskaya et al., 2016).

The reasons for our choice of 5-AZA as a *in-utero* stressor were as follows. First, it was found that 5-AZA at a dose of 0.15 mg/kg does not have teratological effect while still retaining the potential to induce intrauterine stress. Second, our own study (Torchinsky et al., 2012) showed that a single injection of 5-AZA at a sub-threshold teratogenic dose on day 10 of pregnancy triggers loss of femoral trabecular microarchitecture at the young adult age of 5 months (Torchinsky et al., 2012). We also observed that a difference in response to 5-AZA-induced detrimental stimuli between C57 and C3H started early, at the stage of the embryonic limb buds. Whereas 5-AZA treated C3H embryos exhibited a decreased expression of Col1a1, C57 embryos exhibited a decreased expression of Sox9 (Raygorodskaya et al., 2016).

In this study, we further examined whether the long-term effects of an acute teratogenic exposure are still evident in older mice. Mice exposed to a single injection of 5-AZA on day 10 of their mother's pregnancy were evaluated after one year. The main observation of this study is a reduced bone mass in 12-mo-old males of strains C57 and C3H that were exposed to 5-AZA *in-utero*. As expected, we did not find differences in the 3rd lumbar vertebra since *in-utero* exposure to 5-AZA was known to exert bone deficits in the limb buds but not in the axial skeleton. Thus, our studies consistently demonstrated that there is a divergent ability to react to the environmental stimuli, which is dependent on the sex of animal, stronger than on the strain's genetic background, which we keep observing throughout our study. It is also worth noticing that the tested mice exhibited sex-dependent differences in both the character of stress-induced bone abnormalities and age at which the abnormalities were detected. The bones of C3H and C57 mice have similar external size but differ significantly in such traits as adult peak bone density, trabecular thickness, cortical bone density and cortical thickness (Raygorodskaya et al., 2016), as well as biomechanical properties.

It would be important to contemplate why 5-AZA seems to influence appendicular trabecular bone only. Earlier, we (Torchinsky et al., 2012) showed that 5-mo-old offspring of ICR mice exposed to 5-AZA on day 10 of pregnancy exhibited trabecular bone loss. We attribute this to (a) disturbed endochondral ossification mechanisms and (b) higher metabolic activity of the trabecular rather than cortical bone (Clarke, 2008). To wit, long bones develop by endochondral ossification, in which a cartilage template's chondrocytes are replaced by osteoblasts that form the primary spongiosa, which eventually becomes the trabecular bone.(Sakamoto et al., 2005) Later on, both cortical and trabecular bone undergo a continuous process of structural remodeling with the main aim to preserve their biomechanical properties (with cortical component having a prominent role on bone strength); higher metabolic activity of the trabecular bone (Clarke, 2008) makes it more of a subject of loss, even in the early adult age.

In this study, trajectory of changes in bone metrics were calculated as group means and compared between subgroups but not at individual levels. It is worth noticing that the 5-AZA treated C57 male offspring exhibited a moderate bone loss at 3 mo and 6 mo of age (Raygorodskaya et al., 2016) and this detrimental effect was still evident by the age of 12 mo at which these mice had a lower bone mass than the control group (although not statistically significant). In comparison, the 5-AZA exposed C3H male offspring displayed no adverse changes in the femoral microarchitecture at 3 mo of age, whereas they suffered more prominent bone loss at 6 and 12 mo of age. This indicates that the divergent ability of a strain to react to the environmental stimuli depends on the strain's genetic background. Femoral lengths were significantly lower in treated C3H males and females at age 12 months, while no significant changes in BMD or femoral length were observed in treated C57 males and females at this age. Here to note that femoral BMD and femoral length are properties of the whole bone and not necessary reflect the compartmental processes.

Of note, weight in 5-AZA-treated 12-mo-old males of both C3H and C57 strains was significantly lower than that of age-matched controls. Similarly, the weights of treated 12-mo-old C3H and C57 female offspring groups tended to be lower from that of age-matched control groups but the difference was not statistically significant. These results suggest that 5-AZA *in-utero* exposure may have some effect on the body weight of C3H and C57 male and female offspring later in life, and bone length in males can be confounded by their lower body mass. A low body weight is associated with low bone mass (Shapses & Sukumar, 2012), although more so with the areal rather than volumetric BMD. However, mechanisms regulating bone due to weight reduction are not well understood at this time.

We previously reported that strain genetics influences the response to 5-AZA in younger C3H and C57 adult mice (Raygorodskaya et al., 2016). Here we found that in the older individuals, the effects of the strain/genome in response to 5-AZA were weaker than that of the sex. This might be due to a known preponderance of the age-related, rather than genetic, effects on the complex phenotype (van Dongen et al., 2016). Since the cage environment was identical for both strains in our animal facility, this disappearance of the genetic influences can suggest an effect of aging *per se* on the skeleton of 12-mo-old mice. It is also worth noticing that the tested mice exhibited sex-dependent differences in both the character of stress-induced bone abnormalities: 5-AZA-exposed males of both strains had consistently lower femoral length and BMD than age-matched controls, while these differences were milder in females. It is not surprising that there were differences in the response to the intrauterine stressor based on sex. Notable, sex-specific differences were also shown in long-term sequelae of intrauterine inflammation (Dada et al., 2014). Specifically relevant to our stressor, in murine studies, fertility of males treated *in utero* with 5-AZA was more adversely affected compared to females. We thus believe that our study is importantly pointing out the sex-specificity of adverse skeletal outcomes following exposure to teratogen during early development. Since these sex-dependent factors are still unknown, this should be an important direction of the future research.

In summary, the long-term effects of an acute teratogenic exposure are still evident in older mice. Mice exposed to a single injection of 5-AZA on gestational day 10 serve as a model for late-life diseases that were “programmed” by early-life stressors. By characterizing teratogen-exposed C57 and C3H mice, we found that male mice were more sensitive than females to antenatal insults. We therefore confirmed the differences in adaptive responses to antenatal insults between the sexes to remain later in life, although the specific mechanism has to be confirmed with dynamic histomorphometry or similar methods. Findings of this study are relevant for the human health and especially late-life disease such as osteoporosis. In humans, growth pattern during the intrauterine and early-postnatal period is associated with future bone phenotype and fracture risk (reviewed in (Kuh et al., 2014)). Finding molecular mechanisms of the early-life damage should be of help while proposing new therapies to prevent or cure late-life musculoskeletal disease.

The following are the supplementary data related to this article.

Supplementary Fig. 1 Trajectories of change in femoral parameters.
Left panel, males; right panel, females; UT – untreated (control); Tr, treated. Data for 3- and 6-mo mice are from (Raygorodskaya et al., 2016). Image 1

## Transparency document

Transparency document Image 2
