# Supplementary material for: Evaluation of the long-term skeletal effect induced by teratogen 5-aza-2′deoxycytidine on offspring of high (C3H/HeJ) and low (C57BL/6J) bone mass phenotype mice
Source: Bone Rep. 2018 May 29;8:239–43. doi: 10.1016/j.bonr.2018.05.005 (PMC6020399; doi:10.1016/j.bonr.2018.05.005)
Supplement: Supplementary Fig. 1 — Trajectories of change in femoral parameters. Left panel, males; right panel, females; UT – untreated (control); Tr, treated. Data for 3- and 6-mo mice are from (Raygorodskaya et al., 2016). [file mmc1.docx]

Supplementary Figure 1. **Trajectories of change in femoral parameters.**

Left panel, males; right panel, females; UT – untreated (control); Tr, treated. Data for 3- and 6-mo mice are from ^3^.

| **Cortical thickness** |  |
| --- | --- |
|  |  |
| **Trabecular thickness** |  |
|  |  |
| **Trabecular number** |  |
|  |  |
| **Trabecular separation** |  |
|  |  |
| **bone volume/tissue volume ratio** |  |
|  |  |
